# Sustaining the growth of Pinaceae trees under nutrient-limited edaphic conditions via plant-beneficial bacteria

**DOI:** 10.1371/journal.pone.0238055

**Published:** 2020-08-26

**Authors:** Akshit Puri, Kiran Preet Padda, Chris P. Chanway

**Affiliations:** 1 Faculty of Land and Food Systems, The University of British Columbia, Vancouver, British Columbia, Canada; 2 Department of Forest and Conservation Sciences, Faculty of Forestry, The University of British Columbia, Vancouver, British Columbia, Canada; Aarhus University, DENMARK

## Abstract

Lodgepole pine, a prominent Pinaceae tree species native to western North America, is well-known for its ability to thrive in highly disturbed and degraded areas. One such area is the Sub-Boreal Pine-Spruce xeric-cold (SBPSxc) region in British Columbia, Canada, which is characterized by weakly-developed, parched soils that lack an organic forest floor and essential plant-available nutrients. We hypothesized that plant growth-promoting bacteria could play a significant role in sustaining the growth of lodgepole pine trees in the SBPSxc region. Testing this hypothesis, we evaluated plant growth-promoting abilities of six endophytic bacterial strains previously isolated from lodgepole pine trees growing in this region. These bacterial strains significantly enhanced the length and biomass of their natural host (lodgepole pine) as well as a foreign host (hybrid white spruce) in a 540-day long greenhouse trial. This growth stimulation could be linked to the diverse plant growth-promoting (PGP) abilities detected in these strains using *in vitro* assays for inorganic/organic phosphate-solubilization, siderophore production IAA production, ACC deaminase activity, lytic enzymes (chitinase, β-1,3-glucanase, protease, and cellulase) activity, ammonia production and catalase activity. ACC deaminase activity was also detected *in vivo* for all strains using ethylene-sensitive plants–canola and tomato. Notably, strains belonging to the Burkholderiaceae family (HP-S1r, LP-R1r and LP-R2r) showed the greatest potential in all PGP assays and enhanced pine and spruce seedling length and biomass by up to 1.5-fold and 4-fold, respectively. Therefore, such bacterial strains with multifarious PGP abilities could be crucial for survival and growth of lodgepole pine trees in the SBPSxc region and could potentially be utilized as bioinoculant for Pinaceae trees in highly disturbed and nutrient-poor ecosystems.

## Introduction

Boreal ecosystems across the northern hemisphere are home to a wide variety of coniferous trees, mainly belonging to the Pinaceae family such as spruce, pine, fir and cedar. In Canada, one of the largest forested countries in the world, boreal forests cover around 77% of the total forest area [1, 2]. The Sub-Boreal Pine-Spruce xeric-cold (SBPSxc) zone is a nutrient-poor, disturbed boreal region located in the central-interior of British Columbia (BC), a Canadian province with highest timber production rate (42%) [1]. The SBPSxc zone is an extremely dry, cold montane region with a mean annual temperature of 1.7°C and a mean annual precipitation of 389 mm [3]. Soils in this region (as in the rest of BC) are relatively young (10,000 years old) and develop very slowly due to harsh climatic conditions. These soils belong to the Brunisolic soil order according to the Canadian System of Soil Classification (‘Cambisols’ in the World Reference Base for Soil Resources and ‘Inceptisols’ in the US Soil Taxonomy). Soils in this region are sandy (often gravelly) in texture since they develop from granitic rocks and have thin or no organic forest floor with slow mineralization rates [4]. Soils in this region typically have poor physico-chemical health with high carbon to nutrient ratio, low cation exchange capacity, acidic pH, limited organic matter, high bulk density and minimal amounts of plant-available macro- and micro-nutrients [5]. In addition, this region is frequently disturbed by wildfires, logging activity and attack of pests such as the mountain pine beetle, pine blister rust, pine gall rust and pine root collar weevil [3]. Despite such highly disturbed and extreme climatic and edaphic conditions, lodgepole pine (*Pinus contorta* var. *latifolia*)–a prominent member of the Pinaceae family found ubiquitously in Western North America–thrives in the SBPSxc region.

Lodgepole pine has possibly the widest range of environmental tolerance among any coniferous species in North America [6]. It has been reported to thrive under severe soil, moisture and topographical conditions including road cuts, mining sites, fire-affected regions and extremely dry, nutrient-poor sites [7–10]. For instance, lodgepole pine trees were observed to naturally regenerate on a bare gravel substrate without topsoil at highly disturbed mining sites with growth rates similar to lodgepole pine trees growing at nearby undisturbed sites with intact topsoil [8, 9]. Due to the ability of lodgepole pine trees to grow under such stressful environments, some studies have suggested that lodgepole pine trees may form close associations with beneficial microbes including plant-growth-promoting bacteria (PGPB) for their survival and growth [11, 12].

Plant-growth-promoting bacteria have been studied widely in agricultural and horticultural crops for decades [13–16] and several agro-tech companies have made PGPB inoculants commercially available for farmers as an alternative to chemical fertilizers and pesticides [17, 18]. However, the use of such inoculants in natural boreal and temperate forest stands to promote regeneration of coniferous trees is less common, primarily because the association of conifers with PGPB and the underlying mechanisms of tree growth promotion are understudied [19]. Although limited, studies conducted so far have suggested that PGPB, whether residing in the rhizosphere or internal plant tissues (endophytes), could play a crucial role in supporting the growth and health of conifers in the Pinaceae family, such as Douglas-fir (*Pseudotsuga menziesii*), hybrid spruce (*Picea glauca × engelmannii*), lodgepole pine (*Pinus contorta*), white spruce (*Picea glauca*), Engelmann spruce (*Picea engelmannii*), Scots pine (*Pinus sylvestris*), and limber pine (*Pinus flexilis*) [19–22] under a range of environmental conditions. The principal mechanisms by which PGPB can promote plant growth include assisting the plants in obtaining nutrients like nitrogen, phosphorus and iron; modulating the levels of phytohormones like indole-3-acetic acid (IAA) and ethylene to enhance plant growth and overcome abiotic and biotic stresses and; suppressing phytopathogens by degrading their cell walls or by enhancing natural plant defence mechanisms [23]. In a previous study, a lodgepole pine endophyte, *Paenibacillus polymyxa* P2b-2R, was reported to have the ability to produce siderophores to acquire iron, solubilize phosphate, produce IAA and synthesize cell wall degrading enzymes including cellulase and protease [24].

To evaluate if lodgepole pine trees growing in the SBPSxc region form beneficial associations with PGPB to survive on highly nutrient-poor and disturbed soils, we previously isolated 48 potential PGPB strains from the needle, stem, and root tissues of lodgepole pine trees growing at two different sites in the SBPSxc region (52° 00′ 04.2″ N, 124° 59′ 44.7″ W, 1003m a.s.l. and 52° 00′ 09.1″ N, 124° 59′ 25.2″ W, 1035m a.s.l.) [5]. We evaluated the ability of these strains to fix atmospheric nitrogen (a key PGP trait) using acetylene reduction assay and selected six strains that showed potential in this assay [5]. Subsequently, using the ^15^N isotope dilution assay, we observed that these six strains can fix significant amounts of host’s foliar nitrogen (18–50%) from the atmosphere [25]. However, as evidenced in several reports, PGPB can also enhance plant growth via mechanisms other than nitrogen fixation [26–29]. Therefore, we hypothesized that these six strains may stimulate lodgepole pine tree growth in the nutrient-limited SBPSxc region through PGP mechanisms unrelated to nitrogen fixation. To test this hypothesis, we used *in vitro* assays to examine various PGP traits of the six bacterial strains via qualitative and quantitative enzyme assays. We also evaluated the PGP ability of these six bacterial strains *in planta* by inoculating them into their natural host (lodgepole pine) in a 540-day long greenhouse growth trial under nutrient-poor edaphic conditions. In addition, we established a similar greenhouse trial with hybrid white spruce to test the potential of these bacterial strains to promote the growth of another prominent Pinaceae tree species native to the SBPSxc region.

## Materials and methods

### Bacterial strains

As explained above, the six bacterial strains evaluated in this study originated from lodgepole pine trees growing in the nutrient-poor SBPSxc region (52° 00′ 04.2″ N, 124° 59′ 44.7″ W, 1003m a.s.l. and 52° 00′ 09.1″ N, 124° 59′ 25.2″ W, 1035m a.s.l.). We derived antibiotic-resistant mutants of these strains by streaking them multiple times on combined carbon medium (CCM) [30] amended with an antibiotic compound (200 mg/L rifamycin) [31]. The reason for raising such mutants was to track the endophytic and rhizospheric colonization by these bacterial strains in lodgepole pine and hybrid white spruce seedlings during the greenhouse growth trials. Previous studies confirmed that this spontaneous mutation does not affect the PGP efficiency of bacteria [31–34]. The antibiotic-resistant strains were stored in CCM broth amended with 200 mg/L rifamycin and 20% (v/v) glycerol at –80°C. Unless otherwise stated, a frozen culture of each strain was thawed and streaked on CCM agar amended with 200 mg/L rifamycin and incubated for 48 hrs at 30°C before being used in assays.

### Greenhouse growth trials

We established two growth trials (540-day long)–one with lodgepole pine and the other with hybrid white spruce–in the University of British Columbia Plant Care Services’ greenhouse facility. Photosynthetically active radiation of at least 300 μmol/m^2^/s at the canopy level was provided during a 16-hour photoperiod (0600 hrs– 2200 hrs). Since each tree species has a different growth pattern that is not comparable to the other [35], each trial was analyzed separately. In each trial, six bacteria-inoculated and one non-inoculated control treatments were evaluated.

Lodgepole pine and hybrid white spruce seeds that originated from forest stands in the SBPS xc zone in BC (lodgepole pine– 51° 54' N lat., 124° 51' W long., elevation 1320 m; hybrid white spruce– 51° 57' N lat., 124° 59' W long., elevation 1350 m) were obtained from the BC Forest Service Tree Seed Centre, Surrey, BC, Canada. Surface sterilization of seeds was conducted by immersion in 30% (v/v) hydrogen peroxide for 90 s followed by rinsing seeds thrice in sterile distilled water. The effectiveness of surface sterilization was confirmed by imprinting ten randomly selected seeds on tryptic soy agar (TSA) plates which were then incubated for 48 hrs at 30°C to check for surface contamination. Surface-sterilized seeds were stratified using the protocol outlined by the Tree Seed Centre, i.e. storing the seeds aseptically for 28 days at 4°C in sterile cheesecloth bags containing sterile moist silica sand. After the stratification period, ten randomly selected seeds were crushed and imprinted on CCM plates amended with 200 mg/L rifamycin and incubated for 48 hrs at 30°C. The plates were examined for internal seed contamination by any of the six bacterial strains used in this study prior to their inoculation. Three surface-sterilized and stratified seeds of either lodgepole pine or hybrid white spruce were sown aseptically in a Ray Leach Cone-tainer (dia: 38 mm and ht: 210 mm) containing sterile soil growth media (filled to 67% capacity). The soil media consisted of silica sand (69% w/w), Turface (29% w/w) and dolomite (2% w/w), and was fertilized with limited amounts of sterile nutrient solution (20 mL), containing KH_2_PO_4_ (0.14 g/L), MgSO_4_ (0.49 g/L), Ca(NO_3_)_2_ (0.0576 g/L), H_3_BO_3_ (0.001 g/L), MnCl_2_.4H_2_O (0.001 g/L), ZnSO_4_.7H_2_O (0.001 g/L), NaMoO_4_.2H_2_O (0.001 g/L), CuSO_4_.5H_2_O (0.0001 g/L) and Na_2_FeEDTA (0.02 g/L), at the beginning of the growth trials and every 30 days thereafter with a similar solution lacking Ca(NO_3_)_2_. A bacterial suspension (5 mL) of each strain was applied directly over the seeds in each Cone-tainer designated for that strain. The bacterial suspension of each strain was prepared by inoculating a loopful of bacterial growth from CCM plates into CCM broth amended with 200 mg/L rifamycin and agitating the broth at 150 rpm for 48 hrs at 30°C. Bacterial cells were harvested by centrifugation at 3000 xg for 30 min, washed twice in sterile phosphate buffered saline (PBS) (pH 7.4) and resuspended in the same buffer to a density of ca. 10^6^ cfu/mL. Non-inoculated control seeds received 5 mL of sterile PBS. Two weeks after sowing, seedlings were thinned to the single largest germinant per Cone-tainer and were provided with sterile distilled water as required.

The effect of bacterial colonization on the growth of lodgepole pine and hybrid white spruce seedlings was evaluated 540 days after sowing. Ten randomly selected seedlings per tree species from each treatment were analyzed. Seedlings were removed from Cone-tainers and the total seedling length was measured. Seedlings were then oven-dried at 65°C for 48 hrs to determine their dry weight biomass.

Five seedlings per tree species from each treatment were randomly selected to evaluate rhizospheric colonization in lodgepole pine and hybrid white spruce 540 days after sowing. Seedlings were removed from Cone-tainers and gently shaken to remove loosely adhering soil particles from roots. Roots were separated from shoots, placed in Falcon tubes containing 10 mL sterile PBS and vortexed at high setting for 1 min. Serial dilutions were performed and a 0.1 mL aliquot of each dilution was plated on CCM agar amended with 200 mg/L rifamycin. The number of bacterial colonies on each plate were counted after an incubation period of 7 days at 30°C. Ideally, rifamycin should inhibit the growth of all bacteria except the six used in this study since they had acquired antibiotic resistance. Roots were oven-dried for 48 hrs at 65°C to determine their dry weight. Rhizospheric populations were calculated as colony forming units (cfu) per gram of dry root tissue.

Similarly, five seedlings per tree species from each treatment were randomly selected to enumerate the endophytic colonies in internal needle, stem and root tissues of lodgepole pine and hybrid white spruce 540 days after sowing. Seedlings were surface-sterilized by immersing them in 1.3% NaOCl for 5 min and washing them thrice with sterile distilled water. The effectiveness of surface-sterilization was confirmed by imprinting the seedling on TSA plates and incubating the plates for 24 hrs at 30°C to check for surface contamination. Using a mortar and pestle, a sample from each tissue (needle, stem and root) was triturated separately in 1 mL sterile PBS. Serial dilutions of triturated suspensions were performed before plating a 0.1 mL aliquot of each dilution on CCM agar amended with 200 mg/L rifamycin. After incubating the plates for 7 days at 30°C, the number of colonies were counted. The endophytic population in each tissue was calculated as cfu per gram of fresh tissue.

### *In vitro* evaluation of plant growth-promoting mechanisms

#### Phosphorus solubilization, phytate hydrolyzation and siderophore production

The ability to convert inorganic tri-calcium (Ca) phosphate–Ca₃(PO₄)₂ –into more soluble mono- and di-calcium phosphates was evaluated using qualitative plate-based and quantitative broth-based analyses. For the qualitative assay, each bacterial strain was spot-inoculated on Pikovskaya’s (PVK) agar medium plates containing 0.5% tri-Ca phosphate [36]. After incubating the plates for 14 days at 30°C, phosphate-solubilization was determined by the occurrence of a clear halo around bacterial growth. The phosphate-solubilization ability on plates was expressed using the solubilization index [26], where solubilization index (SI) = (halo + colony diameter) / colony diameter. To quantify the amount of phosphate solubilized, each bacterial strain was inoculated in PVK liquid broth to a concentration of 10^6^ cfu/mL and incubated for 72 hrs at 30°C in a shaking incubator (180 rpm). Subsequently, the culture supernatant was obtained via centrifugation for 10 min at 8000x g, and 1 mL of this supernatant was mixed with 500 μL of 10% (w/v) trichloroacetic acid and 4 mL of the colour reagent (1: 1: 1: 2 ratio of 3M H_2_SO_4_: 2.5% (w/v) ammonium molybdate: 10% (w/v) ascorbic acid: distilled water). After incubation for 15 min at room temperature, the absorbance of the resulting solution was measured at 820 nm. The amount of soluble phosphates produced by each strain per mL of the PVK medium (μg/mL) was estimated using a standard KH_2_PO_4_ curve [37].

The ability of each bacterial strain to hydrolyze phytate (an organic form of plant-unavailable phosphate) using the phytase enzyme was evaluated in both qualitative and quantitative assays. For qualitative evaluation, each strain was spot-inoculated onto phytase screening medium (PSM) agar plates containing sodium phytate [38]. After incubating the plates for 14 days at 30°C, phytate hydrolyzation was determined by the development of a clear halo around bacterial growth and was expressed as SI. For quantitative evaluation, each bacterial strain was inoculated to a concentration of 10^6^ cfu/mL in PSM broth and incubated at 30°C for 72 hrs in a shaking incubator (180 rpm). The culture supernatant was then extracted via centrifugation for 15 min at 8000x g and 150 μL of the supernatant was mixed with 600 μL of a solution containing 0.1M Tris-HCl, 2mM sodium phytate and 2mM CaCl_2_. Following the incubation period of 30 min at 37°C, 750 μL of 5% (w/v) trichloroacetic acid and 750 μL of the colour reagent (4: 1 ratio of 1.5% (w/v) ammonium molybdate in 5.5% (v/v) H_2_SO_4_: 2.7% (w/v) ferrous sulfate solution) were added. After 5 min of incubation, the absorbance of the resulting solution was measured at 700 nm. The standard curve of KH_2_PO_4_ was used to estimate the amount of soluble phosphorus released by each bacterial strain by hydrolyzing phytate. One unit (U) of phytase activity was defined as the amount of phytase enzyme required to liberate 1 nmol of soluble phosphorus per minute under the given assay conditions and is expressed per mL of PSM culture [39].

To evaluate siderophore production by the six bacterial strains, each strain was spot-inoculated on chrome azurol S (CAS) agar plates and incubated for 7 days at 30°C [40]. The colour change from blue to orange/deep yellow around the bacterial growth on the CAS agar plates indicated the production of siderophores by bacteria. This area of the orange halo was measured and expressed as cm^2^ [28].

#### IAA production and ACC deaminase enzyme activity

The ability of the six bacterial strains to modulate vital plant hormones (IAA and ethylene) in order to enhance the growth and development of the host plant was analyzed. To evaluate the *in vitro* production of IAA, each bacterial strain (ca. 10^6^ cfu/mL) was inoculated into Luria Bertani broth amended with 5 mM L-tryptophan and incubated for 72 hrs at 28°C in a shaking incubator (150 rpm) [41]. After centrifugation (8000x g; 15 min), 1 mL of culture supernatant was mixed with 100 μL of orthophosphoric acid (10mM) and then 2 mL of the Salkowski’s reagent (1: 30: 50 ratio of 0.5M FeCl_3_: 95% (w/w) sulfuric acid: distilled water) was added. The resulting solution was incubated for 15 min at room temperature and the absorbance of the colour that developed was measured at 530 nm [42]. A standard curve of pure IAA was used to estimate the amount of IAA produced by each strain per mL of the growth medium [43].

The ACC deaminase activity of the six bacterial strains was examined using *in vitro* and *in vivo* techniques described by Penrose and Glick [44]. Each strain was grown to stationary phase in tryptic soy broth (nutrient-rich medium) at 30 ºC. Bacterial cells of each strain were harvested via centrifugation at 8000x g. To induce the ACC deaminase activity, bacterial cells were suspended in DF salts minimal medium (nutrient-poor medium) amended with 3mM ACC as the sole source of nitrogen and grown for 24 hrs at 30 ºC in a shaking incubator (200 rpm). The bacterial cells were harvested via centrifugation (8000x g), washed and suspended either in 0.1M Tris-HCl for *in vitro* analysis or in 0.03M MgSO_4_ for *in vivo* analysis. For the *in vitro* assay, bacterial cells suspended in 0.1M Tris-HCl were mixed with toluene, and a portion of the toluene-treated cells was mixed with 0.5M ACC and incubated for 15 min at 30 ºC. After adding 0.56M HCl, the solution was mixed, and the supernatant was collected by centrifugation (16000x g). The supernatant was then mixed with 0.56M HCl and 2,4-dinitrophenylhydrazine reagent (0.2% 2,4-dinitrophenylhydrazine in 2M HCl) and incubated for 30 min at 30 ºC. After adding 2M NaOH, the absorbance of the resulting solution was measured at 540 nm. ACC deaminase activity was quantified using pure α-ketobutyrate as the standard and expressed as the amount of α-ketobutyrate produced per mg protein per hour. To analyze ACC deaminase activity *in vivo*, a gnotobiotic root elongation assay was used involving ethylene-sensitive plants–canola (*Brassica napus*) and tomato (*Solanum lycopersicum*). Canola seeds (var. *Rugby Roundup ready*) were obtained from the SeCan Association’s Alberta branch (Lamont, AB, Canada). Tomato seeds (var. *Celebrity*) were obtained from the West Coast Seed Company, Delta, BC, Canada. For surface-sterilization, seeds were immersed in 30% hydrogen peroxide for 90 s and washed thrice in sterile distilled water. Effectiveness of the surface sterilization was confirmed by imprinting ten randomly selected canola and tomato seeds on tryptic soy agar (TSA) plates which were incubated for 48 hrs at 30°C to check for surface contamination. The ACC-induced bacterial cells of each strain suspended in 0.03M MgSO_4_ (OD_600_ = 0.15) were used in this assay. Surface-sterilized canola and tomato seeds were incubated in petri dishes for 1 hr with one of the following treatments: sterile 0.03M MgSO_4_ (control) or bacterial suspensions of each of the six strains. Following the incubation period, 7 seeds per plant species from each treatment were aseptically placed in sterile CYG™ germination pouches (Mega International, Newport, MN, USA) containing 15 mL sterile distilled water. Subsequently, the pouches were incubated in a growth chamber (Conviron CMP3244, Conviron Products Company, Winnipeg, MB, Canada) maintained at 20 ºC with a day/night cycle beginning with 12 hrs of dark followed by 12 hrs of light, with light intensity set to 18 μmol/m^2^/s. The primary root lengths of canola and tomato seedlings from each treatment were measured five days after germination.

#### Activity of cell wall degrading enzymes

The ability of the six bacterial strains to secrete key cell wall degrading enzymes (chitinase, β-1,3-glucanase, protease and cellulase) was evaluated *in vitro*. Qualitative evaluation of chitinase activity included spot-inoculating each strain on chitin agar plates containing 1.62 g nutrient broth (Sigma-Aldrich, USA), 0.5 g NaCl, 6 g M 9 salts (Difco, USA), 8 g colloidal chitin and 15 g agar per litre [45]. After incubation for 7 days at 30°C, a clear halo surrounding the bacterial growth indicated positive chitinase activity and the width of the clearance zone was calculated as = (halo + colony diameter)–(colony diameter). The amount of colloidal chitin converted to simple sugars due to the chitinase activity was quantified by inoculating each bacterial strain to a concentration of 10^6^ cfu/mL in liquid chitin medium. After incubating for 5 days at 30°C in a shaking incubator (150 rpm), the culture supernatant was separated by centrifugation for 15 min at 8000x g and 500 μL of the supernatant was mixed with 500 μL of 1 M phosphate buffer and 500 μL of colloidal chitin solution containing 10 mg chitin. The resulting solution was incubated for 30 min at 37°C and centrifuged for 3 min at 8000x g to collect the supernatant. The supernatant (1 mL) was mixed with dinitrosalicylic acid (2 mL) and heated for 5 min in a boiling water bath. The absorbance of the final solution was measured at 575 nm [37]. Using glucose as the standard, the chitinase enzyme activity was estimated by measuring the release of reducing sugars from chitin. One unit (U) of chitinase activity was defined as the amount of chitinase enzyme that resulted in the release of 1 μmol of glucose from colloidal chitin per minute.

The β-1,3-glucanase activity was evaluated qualitatively by spot-inoculating each bacterial strain on plates containing β-1,3-glucan (laminarin) as the sole carbon source (5 g/L) along with other essential nutrients outlined by Renwick et al. [46]. Following incubation for 3 days at 30°C, plates were stained with Congo Re d (0.6 g/L) and left at room temperature for 90 min. The hydrolysis of glucan (i.e. glucanase activity) was indicated by the development of a yellow/orange zone around the bacterial growth on plates and the width of this yellow/orange zone was measured. For the quantitative determination of β-1,3-glucanase activity, the aforementioned laminarin medium (without agar) was inoculated with each bacterial strain (ca. 10^6^ cfu/mL) and incubated for 5 days at 30°C in a shaking incubator (150 rpm). The culture supernatant was extracted by centrifugation for 15 min at 8000x g and 500 μL of the supernatant was mixed with 500 μL of 1 M citrate buffer (pH 5.0) and 500 μL of 4% laminarin. After an incubation period of 30 min at 37°C, 2 mL of dinitrosalicylic acid was added and the solution was heated for 5 min in a boiling water bath. The absorbance of the resulting solution was measured at 500 nm [37]. The β-1,3-glucanase activity was estimated by measuring the release of reducing sugars from laminarin using glucose as the standard. One unit (U) of β-1,3-glucanase activity was defined as the amount of β-1,3-glucanase enzyme that resulted in the release of 1 μmol of glucose from laminarin per minute.

To evaluate the protease enzyme activity qualitatively, each bacterial strain was spot-inoculated on casein–yeast extract (CYE) agar plates amended with 7% skimmed milk powder [24]. The plates were incubated for 7 days at 30°C, and the development of a clear zone surrounding the bacterial growth indicated protease activity. The width of the clear zone was measured. To quantify the protease enzyme activity, each bacterial strain was inoculated to a concentration of 10^6^ cfu/mL in CYE liquid medium amended with 7% skimmed milk powder and incubated for 5 days at 30°C in a shaking incubator (150 rpm). The culture supernatant was extracted by centrifugation for 15 min at 8000x g and 500 μL of the supernatant was mixed with 500 μL of 0.2 M phosphate buffer and 500 μL of 1% azocasein. After incubation for 30 min at 37°C, 2 mL of 10% (w/v) trichloroacetic acid was added and the solution was further incubated for 5 min at room temperature. The absorbance was measured at 440 nm after the addition of 1M NaOH (1mL). Protease activity was determined by measuring the release of reducing amino acids from azocasein using tyrosine as the standard. One unit of protease enzyme activity was defined as the amount of the protease enzyme that resulted in the release of 1 μmol of tyrosine from azocasein per minute [37].

The cellulase enzyme activity for each bacterial strain was assessed qualitatively by spot-inoculating on CYE agar plates amended with 1% sodium carboxymethylcellulose [47]. After incubating for 48 hrs at 30°C, the plates were flooded with Congo red solution (0.5% w/v) and left at room temperature for 30 min. Subsequently, plates were drained and rinsed with 1 mol/L NaCl and the development of a clear zone around the bacterial growth indicated cellulase activity. The width of the clearance zone was measured. For quantitative evaluation of cellulase activity, each strain was inoculated to a concentration of 10^6^ cfu/mL in CYE liquid medium amended with 1% sodium carboxymethylcellulose and incubated for 5 days at 30°C in a shaking incubator (150 rpm). The culture supernatant was extracted via centrifugation for 15 min at 8000x g and 500 μL of the supernatant was mixed with 500 μL of 1M citrate buffer and 500 μL of 1% carboxymethylcellulose. After an incubation period of 30 min at 37°C, dinitrosalicylic acid (2 mL) was added and the solution was heated for 5 min in a boiling water bath. The absorbance of the resulting mixture was measured at 500 nm [45]. Using glucose as the standard, cellulase activity was estimated by measuring the release of reducing sugars from carboxymethylcellulose. One unit (U) of cellulase enzyme activity was defined as the amount of cellulase enzyme that resulted in the release of 1 μmol of glucose from carboxymethylcellulose per minute [37].

#### Ammonia production

The six bacterial strains were analyzed for *in vitro* production of ammonia by inoculating each strain (ca. 10^6^ cfu/mL) into 10 mL of peptone water and incubating for 72 hrs at 30°C. After that, 500 μL of Nessler’s reagent was added and the development of a brown-yellow colour indicated ammonia production [48].

#### Catalase enzyme activity

Catalase activity was evaluated for each bacterial strain by mixing a loopful of fresh bacterial culture with 50 μL of 3% (v/v) hydrogen peroxide on a sterile glass slide and incubating at room temperature for 1 min. The evolution of oxygen, i.e. development of gas bubbles, indicated a positive catalase reaction [24].

### Statistical analyses

Each plant growth trial was arranged in a completely randomized experimental design with 20 replicates per treatment (5 replicates to evaluate rhizospheric colonization; 5 replicates to evaluate endophytic colonization and; 10 replicates to evaluate seedling length and biomass). Statistical analyses for each greenhouse trial (one involving lodgepole pine and the other involving hybrid white spruce) were performed separately since each tree species has a different growth pattern, not comparable to one another [35]. Analysis of variance (ANOVA) was performed (F-test and Student’s t-test) to determine significant differences between treatment means for seedling length and seedling biomass in each trial. ANOVA was also performed to determine differences between treatment means for the quantitative assays evaluating phosphate solubilization, phytate hydrolyzation, ACC deaminase activity, IAA production, cellulase activity, protease activity, chitinase activity and β-1,3-glucanase activity as well as for siderophore production and root elongation in the gnotobiotic assay. Three replicates per treatment were evaluated for all quantitative assays as well as for siderophore production, and seven replicates per treatment were evaluated for the gnotobiotic root elongation assay. The statistical package, SAS University Edition (SAS Institute Inc., Cary, NC, USA), was used to perform all statistical analyses (α = 0.05).

## Results

### Greenhouse growth trials

**Fig 1:**
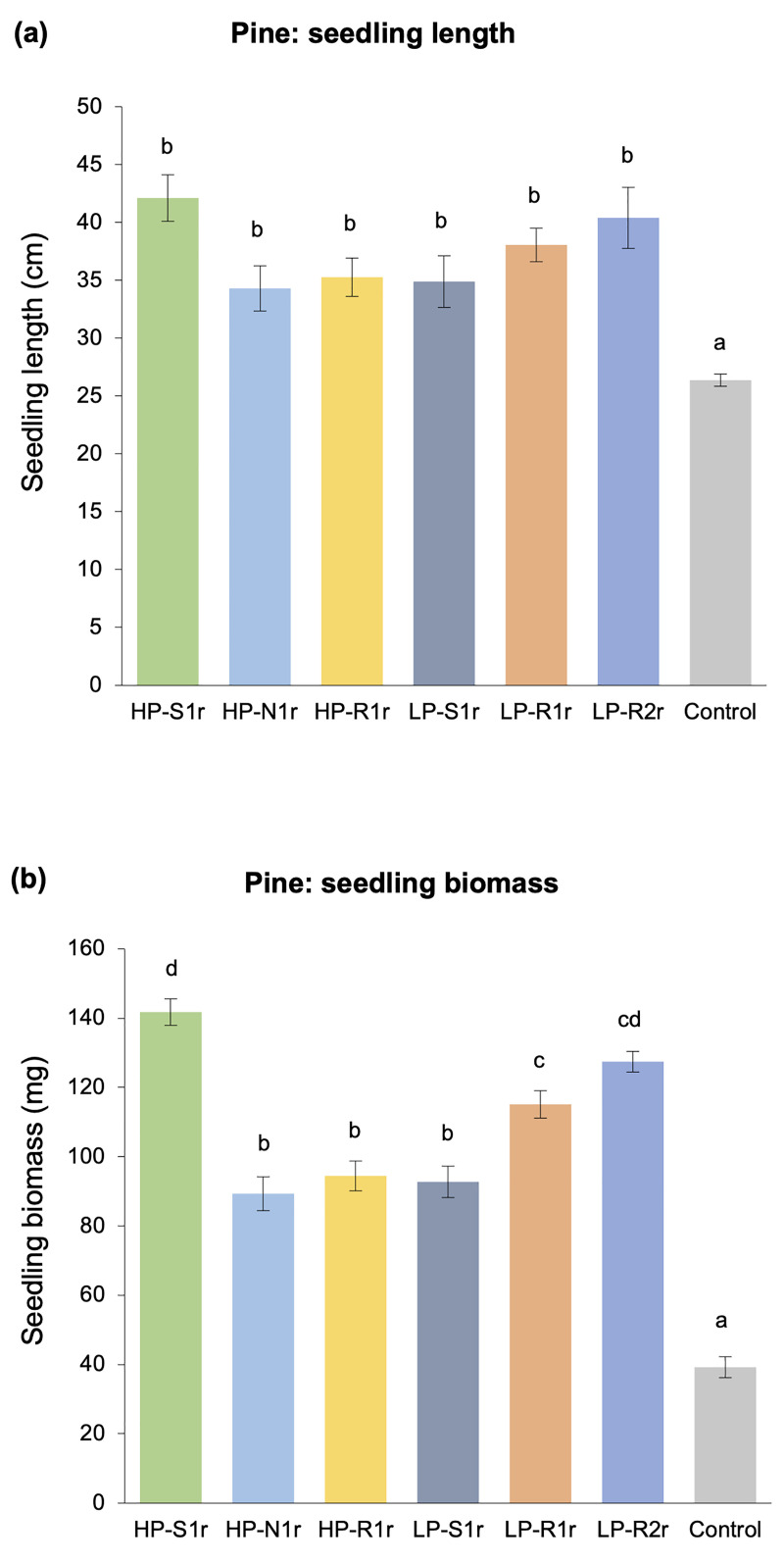
Mean values of (a) length and (b) biomass of 540-day old lodgepole pine seedlings subjected to six bacteria-inoculated (*Caballeronia sordidicola* HP-S1r, *Pseudomonas frederiksbergensis* HP-N1r, *Phyllobacterium myrsinacearum* HP-R1r, *Pseudomonas mandelii* LP-S1r, *Paraburkholderia phytofirmans* LP-R1r and *Caballeronia udeis* LP-R2r) and one non-inoculated control treatments. Error bars represent standard errors of the mean (n = 10 seedlings per treatment) and bars with different letters are significantly different (P < 0.05).

**Fig 2:**
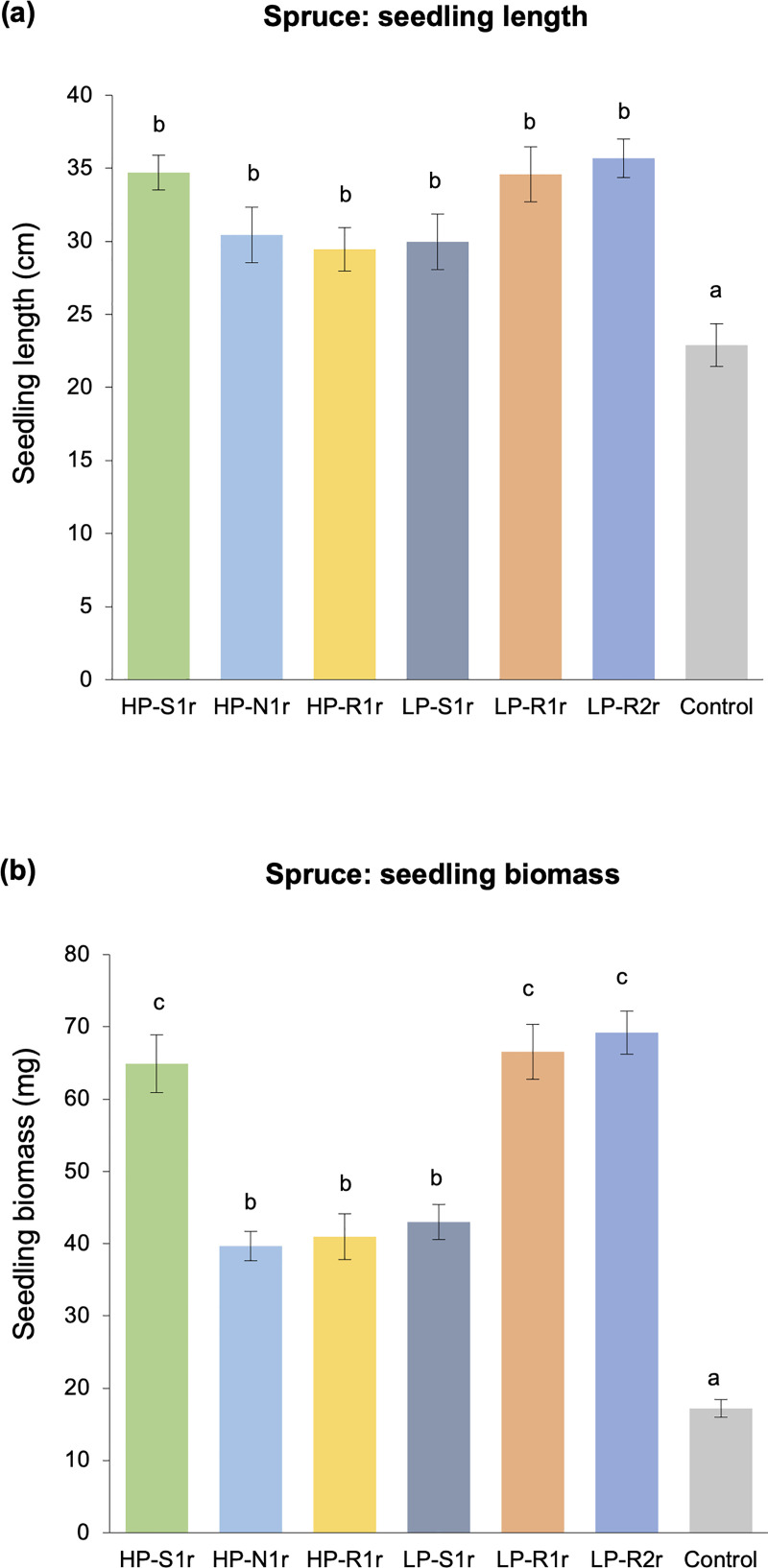
Mean values of (a) length and (b) biomass of 540-day old hybrid white spruce seedlings subjected to six bacteria-inoculated (*Caballeronia sordidicola* HP-S1r, *Pseudomonas frederiksbergensis* HP-N1r, *Phyllobacterium myrsinacearum* HP-R1r, *Pseudomonas mandelii* LP-S1r, *Paraburkholderia phytofirmans* LP-R1r and *Caballeronia udeis* LP-R2r) and one non-inoculated control treatments. Error bars represent standard errors of the mean (n = 10 seedlings per treatment) and bars with different letters are significantly different (P < 0.05).

Each of the six bacterial strains analyzed in this study had a significant positive effect on the growth of 540-day old lodgepole pine and hybrid white spruce seedlings. All bacterial treatments significantly promoted the length and biomass of pine and spruce seedlings as compared to the non-inoculated control treatment (Figs 1 and 2). Bacteria-inoculated pine seedlings had > 125% higher biomass (on average) and were > 30% longer (on average) than the non-inoculated control seedlings (Fig 1). Similarly, spruce seedlings inoculated with bacteria were also > 30% longer and had > 130% higher biomass than the control seedlings (Fig 2). Notably, the pine and spruce seedlings inoculated with strains *Caballeronia sordidicola* HP-S1r, *Paraburkholderia phytofirmans* LP-R1r and *Caballeronia udeis* LP-R2r were around 50% longer than controls (Figs 1A and 2A). Moreover, inoculation with these three strains enhanced pine seedling biomass by > 200% (Fig 1B) and spruce seedling biomass by > 275% (Fig 2B) as compared to the control.

**Fig 3:**
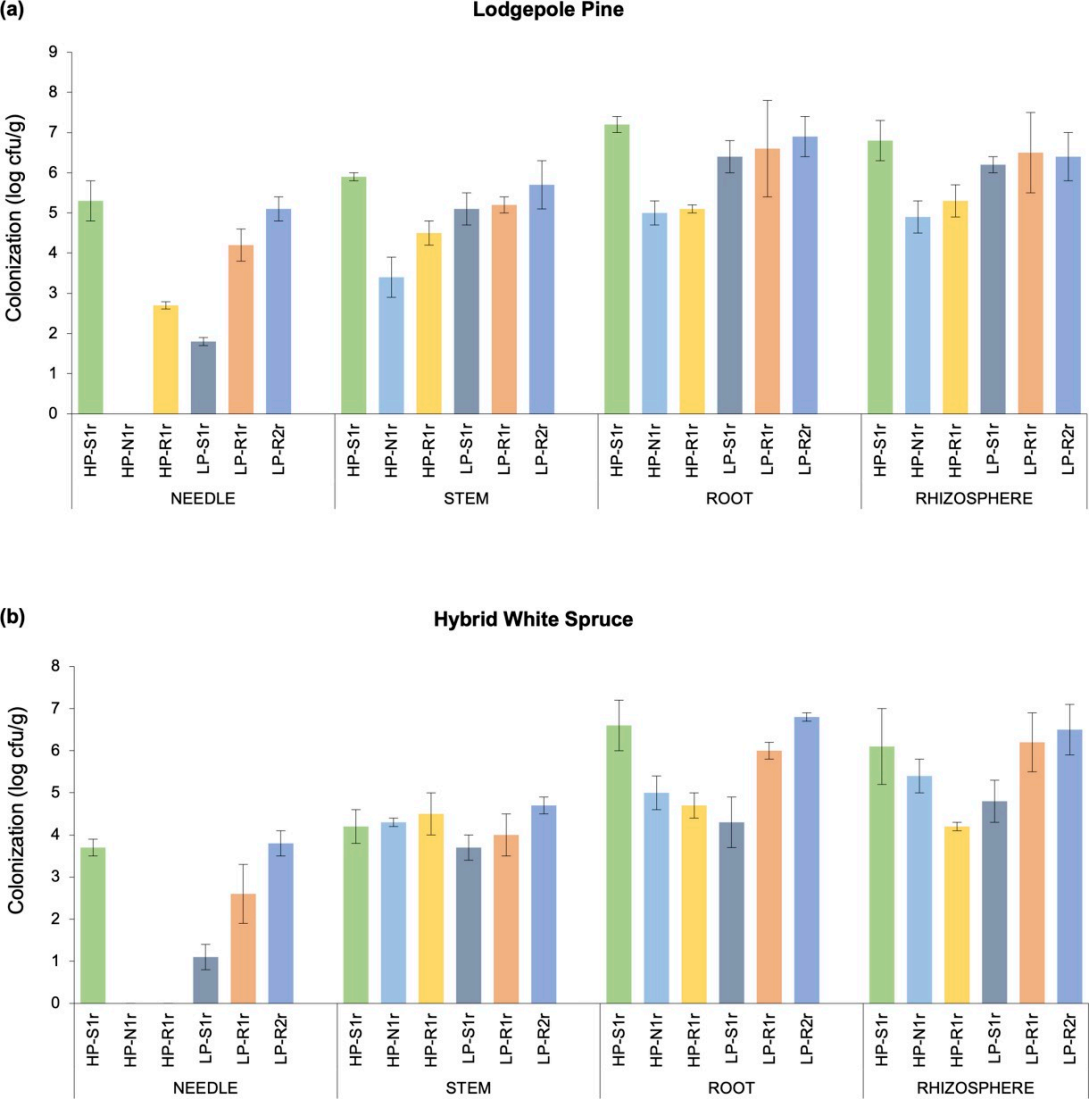
Population density of each of the six bacterial strains (*Caballeronia sordidicola* HP-S1r, *Pseudomonas frederiksbergensis* HP-N1r, *Phyllobacterium myrsinacearum* HP-R1r, *Pseudomonas mandelii* LP-S1r, *Paraburkholderia phytofirmans* LP-R1r and *Caballeronia udeis* LP-R2r) inside the endophytic tissues (needle, stem and root) and in the rhizosphere of (a) lodgepole pine and (b) hybrid white spruce seedlings evaluated 540 days after inoculation. For clarity of presentation, the data was log-transformed. Error bars represent standard errors of the mean (n = 5 seedlings per treatment for endophytic colonization and 5 seedlings per treatment for rhizospheric colonization).

**Fig 4:**
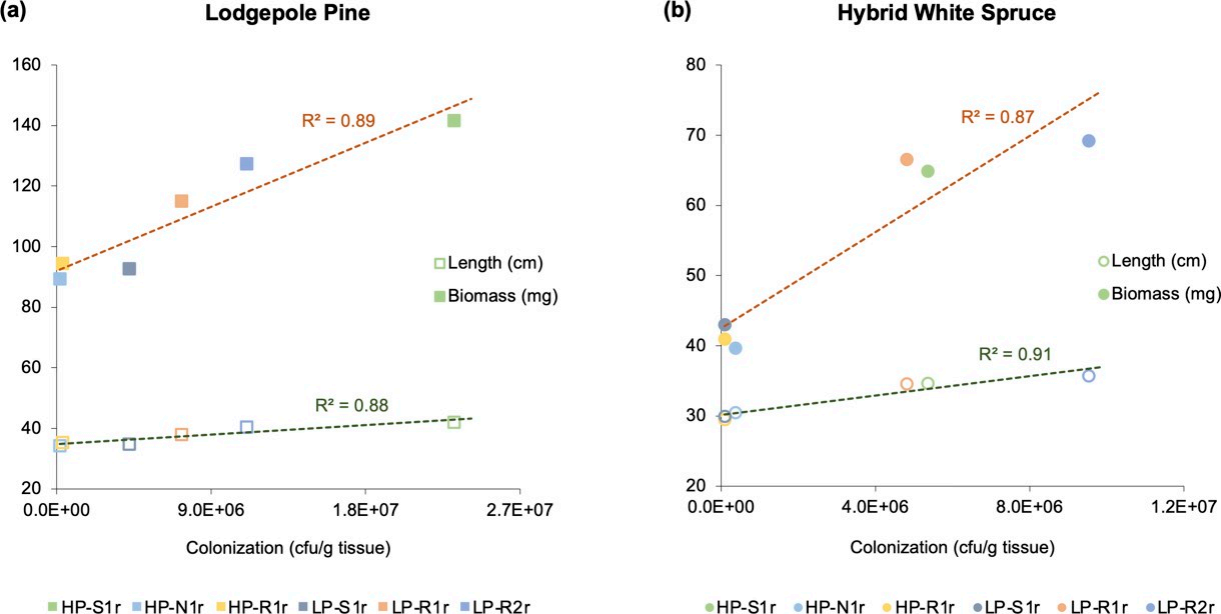
Correlation of bacterial population sizes (x-axis) with length and biomass (y-axis) of (a) lodgepole pine and (b) hybrid white spruce seedlings. For this correlation, the population sizes in the rhizosphere and internal tissues were aggerated for each strain (*Caballeronia sordidicola* HP-S1r, *Pseudomonas frederiksbergensis* HP-N1r, *Phyllobacterium myrsinacearum* HP-R1r, *Pseudomonas mandelii* LP-S1r, *Paraburkholderia phytofirmans* LP-R1r and *Caballeronia udeis* LP-R2r). Population size, length and biomass variables were analyzed 540 days after the inoculation of pine and spruce.

Each of the six bacterial strains evaluated in this study had the ability to form rhizospheric and endophytic colonies in their original host (lodgepole pine) as well as the foreign host (hybrid white spruce). The bacterial strains, on average, had population sizes of 10^4^–10^6^ cfu/g dry root tissue in the rhizosphere of pine and spruce seedlings, 540 days after inoculation (Fig 3). Similar population sizes were observed in the internal root tissues of pine and spruce seedlings. All strains were able to colonize the stem tissues with population sizes ranging from 10^3^ to 10^5^ cfu/g tissue in pine and 10^3^ to 10^4^ cfu/g tissue in spruce (Fig 3). Needle colonization was observed for all strains except *Pseudomonas frederiksbergensis* HP-N1r in pine seedlings, with population densities ranging between 10^1^ and 10^5^ cfu/g tissue (Fig 3A). Spruce needles were colonized by four bacterial strains only (10^1^–10^3^ cfu/g tissue) as no colonies were observed in the needle tissues of *P*. *frederiksbergensis* HP-N1r and *Phyllobacterium myrsinacearum* HP-R1r inoculated spruce seedlings (Fig 3B). Strains *C*. *sordidicola* HP-S1r and *C*. *udeis* LP-R2r showed the highest colonization potential in stem and needle tissues of pine and spruce (up to 10^5^ cfu/g tissue). Moreover, these two strains along with strain *P*. *phytofirmans* LP-R1r colonized the root tissues and rhizosphere of pine and spruce extensively (10^6^–10^7^ cfu/g tissue). No bacterial colonies were observed in the rhizosphere and internal tissues of non-inoculated control pine and spruce seedlings. When the number of colonies formed by each bacterial strain outside and inside pine and spruce seedlings were aggregated and compared with the length and biomass variables, a strong correlation was observed (R^2^ > 0.87) (Fig 4).

### In vitro evaluation of plant growth-promoting mechanisms

The ability of bacterial strains to convert inorganic tri-Ca phosphate and organic phytate into soluble phosphate forms was observed for all strains except *P*. *frederiksbergensis* HP-N1r (Tables 1 and 2). The SI for phosphate ranged from 1.2 to 2.6 whereas for phytate hydrolysis it ranged from 2.0 to 3.6 (Table 1). These results were consistent with the quantitative results since the same bacterial strains solubilized phosphate and phytate in broth-based assays (Table 2). The amount of soluble phosphates detected in the tri-Ca phosphate solubilization test ranged between 66 and 112 μg/mL (Table 2). For the phytate hydrolyzation test, 42–86 units of phytase enzyme were detected per mL of the broth (Table 2). Strain *C*. *udeis* LP-R2r solubilized > 2.5 times tri-Ca phosphate and > 3.5 times phytate relative to its growth on plates. In addition, this strain solubilized significantly higher amounts of phosphate (up to 68%) and secreted significantly greater amounts of phytase (up to 103%) than all other bacterial strains in the broth assays. Strains *C*. *sordidicola* HP-S1r and *P*. *phytofirmans* LP-R1r also showed considerable phosphate solubilization and phytate hydrolyzation activities in both qualitative and quantitative evaluations (Tables 1 and 2). The ability to produce siderophores was observed for all strains except *P*. *myrsinacearum* HP-R1r on the CAS blue agar. The area of the orange halo produced by the bacterial strains on CAS agar plates ranged from 1.63 to 2.73 cm^2^, with strain *C*. *udeis* LP-R2r producing the largest orange halo closely followed by strain *P*. *phytofirmans* LP-R1r (Table 1).

**Table 1:** Qualitative evaluation of major direct and indirect plant-growth-promoting mechanisms of the six bacterial strains using *in vitro* plate-based enzyme assays.

Bacterial strains	Phosphate solubilization (SI) ^†^	Phytate hydrolyzation (SI) ^†^	Siderophore production (area of orange halo in cm^2^) ^‡^	Chitinase activity ^§^	β-1,3-glucanase activity ^§^	Protease activity ^§^	Cellulase activity ^§^	Ammonia production ^#^	Catalase activity ^#^
*Caballeronia sordidicola* HP-S1r	1.5	3.2	1.93 ± 0.09^ab^	+++	++	+	+	+	+
*Pseudomonas frederiksbergensis* HP-N1r	–	–	2.33 ± 0.12^bc^	–	–	+	–	+	–
*Phyllobacterium myrsinacearum* HP-R1r	1.2	2	–	–	–	–	+	+	++
*Pseudomonas mandelii* LP-S1r	1.3	2.2	1.63 ± 0.09^a^	+	–	+	++	++	++
*Paraburkholderia phytofirmans* LP-R1r	1.9	2.9	2.43 ± 0.12^bc^	++	+	+++	+++	+++	+
*Caballeronia udeis* LP-R2r	2.6	3.6	2.73 ± 0.10^c^	+	+++	++	+++	+	+++

^†^ solubilization index (SI) represents the zone of solubilization on triplicate plates relative to bacterial growth.

^‡^ values are mean ± standard error (n = 3), where values followed by different letters are significantly different at P < 0.05.

^§^ clearance zone for chitinase, protease and cellulase activities and yellow-orange zone for β-1,3-glucanase activity was evaluated for each bacterial strain on triplicate plates, where ‘–’ means no observed zone, ‘+’ means 0–5 mm zone, ‘++’ means 5–15 mm zone, and ‘+++’ means 15–25 mm zone.

^#^ assessed using triplicate samples per bacterial strain, where ‘–’ means no production/activity, ‘+’ means low production/activity, ‘++’ means medium production/activity and ‘+++’ means high production/activity.

**Table 2:** Quantitative evaluation of major direct and indirect plant-growth-promoting mechanisms of the six bacterial strains using *in vitro* broth-based enzyme assays.

Bacterial strains	Phosphate solubilization (μg/mL) ^†^	Phytate hydrolyzation (U/mL) ^†^	IAA production (μg/mL) ^†^	ACC deaminase activity (nmol α-ketobutyrate/mg/hr) ^†^	Chitinase activity (U/mL) ^†^	β-1,3-glucanase activity (U/mL) ^†^	Protease activity (U/mL) ^†^	Cellulase activity (U/mL) ^†^
*Caballeronia sordidicola* HP-S1r	96.3 ± 0.88^b^	79.5 ± 0.45^c^	32.0 ± 0.83^c^	101 ± 1.17^d^	0.52 ± 0.02^c^	0.59 ± 0.02^a^	51.8 ± 0.42^c^	0.35 ± 0.02^b^
*Pseudomonas frederiksbergensis* HP-N1r	–	–	21.0 ± 0.92^b^	33.3 ± 0.69^a^	–	–	37.3 ± 1.45^b^	–
*Phyllobacterium myrsinacearum* HP-R1r	66.7 ± 1.20^a^	43.0 ± 0.38^a^	15.5 ± 0.69^a^	65.8 ± 1.03^c^	–	–	–	0.17 ± 0.01^a^
*Pseudomonas mandelii* LP-S1r	70.3 ± 1.45^a^	42.7 ± 0.55^a^	23.5 ± 0.69^b^	54.4 ± 1.16^b^	0.28 ± 0.01^a^	–	20.3 ± 1.45^a^	0.51 ± 0.02^c^
*Paraburkholderia phytofirmans* LP-R1r	92.7 ± 1.76^b^	68.2 ± 0.42^b^	29.8 ± 0.91^c^	96.3 ± 1.26^d^	0.49 ± 0.02^c^	0.51 ± 0.02^a^	82.3 ± 1.45^e^	0.54 ± 0.01^cd^
*Caballeronia udeis* LP-R2r	112 ± 1.45^c^	86.6 ± 0.23^d^	32.3 ± 0.55^c^	111 ± 1.41^e^	0.40 ± 0.01^b^	0.89 ± 0.01^b^	73.4 ± 0.33^d^	0.61 ± 0.02^d^

^†^ Values are mean ± standard error (n = 3), where values followed by different letters are significantly different (P < 0.05).

**Fig 5:**
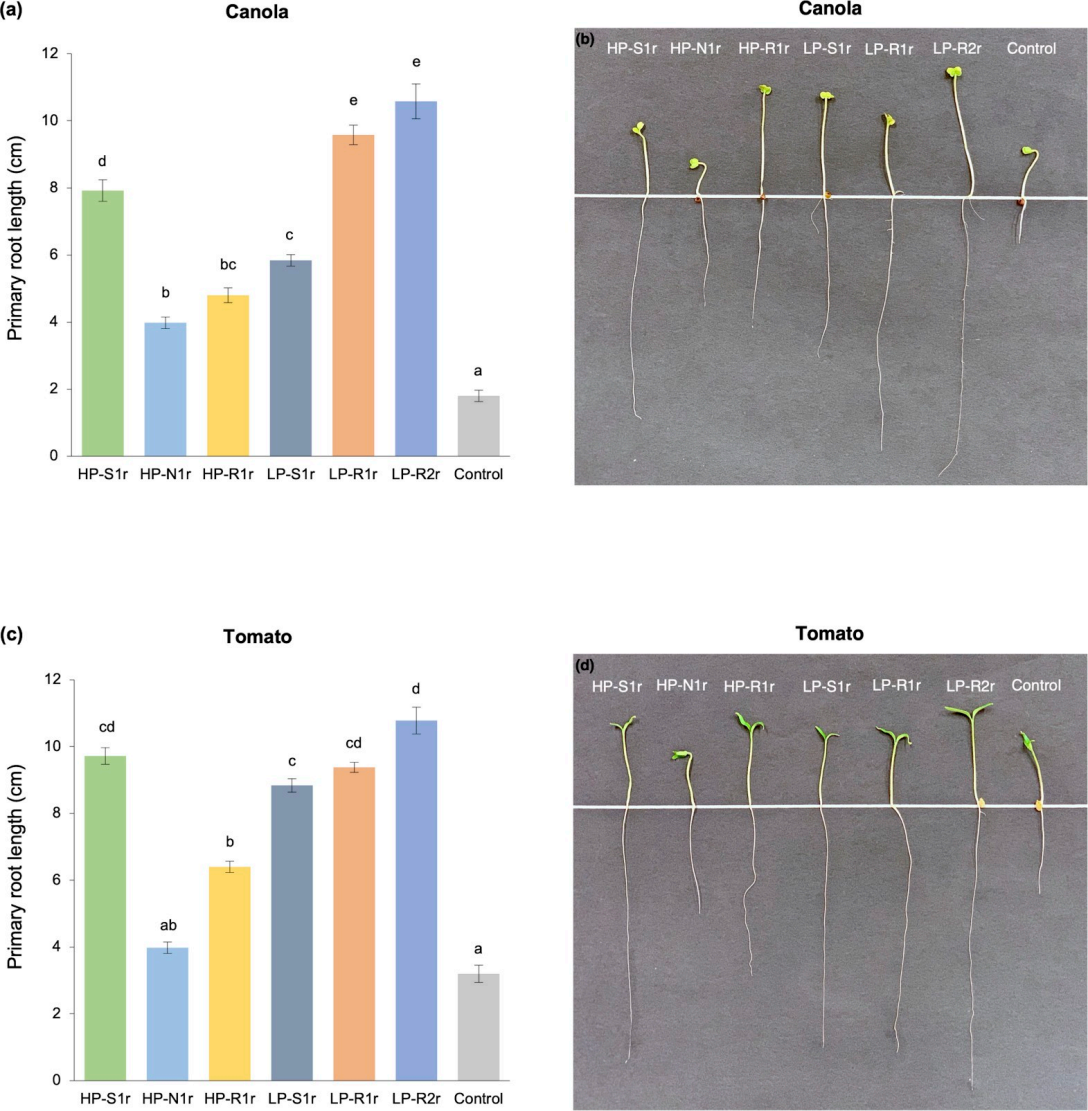
Primary root length of (a), (b) canola and (c), (d) tomato seedlings subjected to six bacteria-inoculated (*Caballeronia sordidicola* HP-S1r, *Pseudomonas frederiksbergensis* HP-N1r, *Phyllobacterium myrsinacearum* HP-R1r, *Pseudomonas mandelii* LP-S1r, *Paraburkholderia phytofirmans* LP-R1r and *Caballeronia udeis* LP-R2r) and one non-inoculated control treatments. Seedlings were evaluated five days after germination in the gnotobiotic root elongation assay to evaluate *in situ* ACC deaminase activity. Error bars represent standard errors of the mean (n = 7 seedlings per treatment) and bars with different letters are significantly different (P < 0.05).

**Fig 6:**
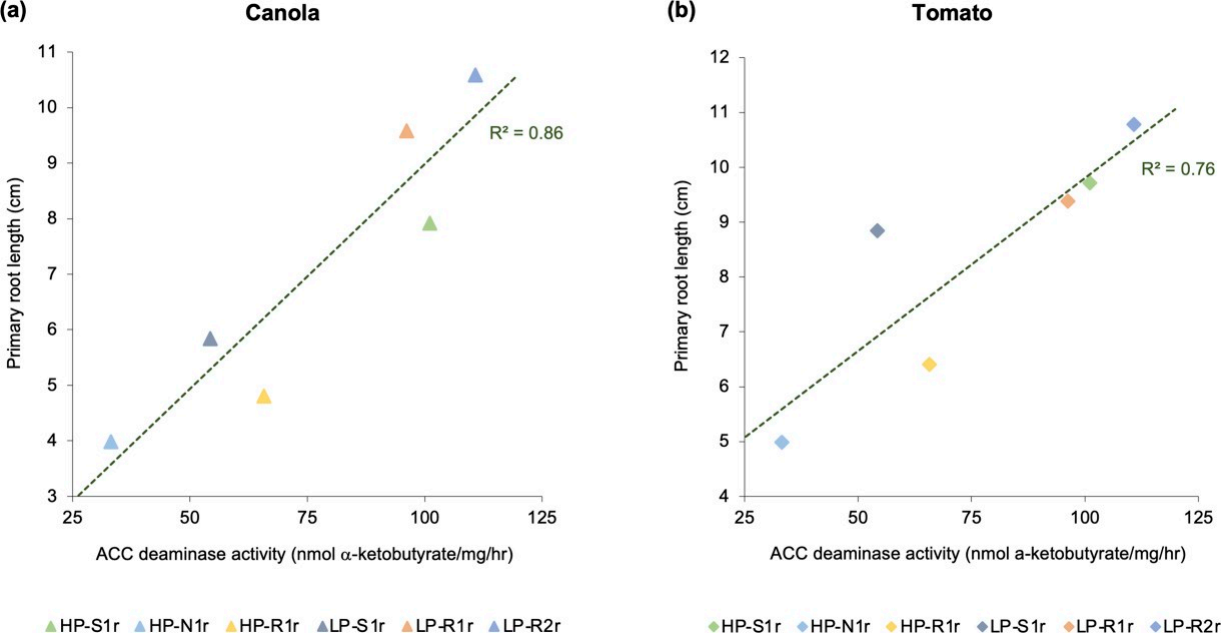
Correlation of *in vitro* ACC deaminase activity (x-axis) with primary root length (y-axis) of (a) canola and (b) tomato seedlings. The *in vitro* ACC deaminase activity was measured as the amount of α-ketobutyrate produced per mg protein in an hour. The primary root length of canola and tomato enhanced by each bacterial strain (*Caballeronia sordidicola* HP-S1r, *Pseudomonas frederiksbergensis* HP-N1r, *Phyllobacterium myrsinacearum* HP-R1r, *Pseudomonas mandelii* LP-S1r, *Paraburkholderia phytofirmans* LP-R1r and *Caballeronia udeis* LP-R2r)reflects the *in vivo* ACC deaminase activity.

All bacterial strains were capable of producing IAA and ACC deaminase *in vitro*. The amount of IAA produced from L-tryptophan by bacterial strains ranged from 15 to 32 μg/mL of broth, with strains *C*. *udeis* LP-R2r, *C*. *sordidicola* HP-S1r and *P*. *phytofirmans* LP-R1r producing significantly higher IAA than other bacterial strains (Table 2). The amount of α-ketobutyrate produced (when ACC deaminase enzyme cleaves ACC) by strains ranged between 33 and 111 nmol per mg of protein in an hour (Table 2). Notably, strain *C*. *udeis* LP-R2r produced a significantly higher amount of α-ketobutyrate than all other strains. In addition, strains *C*. *sordidicola* HP-S1r and *P*. *phytofirmans* LP-R1r also produced considerable amounts of α-ketobutyrate in this assay. Similar ACC deaminase activity was observed in the plant-based gnotobiotic assay. All bacterial strains significantly enhanced the root length of canola and tomato plants in the gnotobiotic assay by synthesizing ACC deaminase to suppress the overproduction of plant-produced ethylene after germination. The primary root length of five-day-old bacteria-inoculated canola and tomato plants was more than 2-fold greater compared to control plants (Fig 5). Notably, canola and tomato plants inoculated with strain *C*. *udeis* LP-R2r had the longest primary roots of all treatments (487% and 237%, respectively greater than controls). In addition, strains *C*. *sordidicola* HP-S1r and *P*. *phytofirmans* LP-R1r promoted canola root length by 340% and 432%, respectively, and tomato root length by 204% and 193%, respectively (Fig 5). The primary root length of both canola and tomato seedlings inoculated with bacterial strains had a strong correlation (R^2^ > 0.75) with the amount of α-ketobutyrate produced by these strains (Fig 6). In particular, *C*. *sordidicola* HS-S1r, *P*. *phytofirmans* LP-R1r and *C*. *udeis* LP-R2r strains that produced the highest amounts of α-ketobutyrate, also showed highest root length enhancement for both canola (up to 6-fold) and tomato (up to 3-fold) (Fig 6).

All six bacterial strains were able to produce at least one of the four major cell wall degrading enzymes (chitinase, β-1,3-glucanase, protease and cellulase). Chitinase activity was observed in four strains in both qualitative plate-based and quantitative broth-based assays but strains *P*. *frederiksbergensis* HP-N1r and *P*. *myrsinacearum* HP-R1r showed no activity (Tables 1 and 2). Strain *C*. *sordidicola* HP-S1r showed the greatest chitinase activity, solubilizing around 15–25 mm of the colloidal chitin relative to its growth in the plate assay and producing around 0.52 units of chitinase enzyme in the broth assay. The β-1,3-glucanase activity was detected in three strains only (*C*. *sordidicola* HP-S1r, *P*. *phytofirmans* LP-R1r and *C*. *udeis* LP-R2r) in the qualitative plate-based assay, which was consistent with the results of the quantitative broth-based assay (Tables 1 and 2). Strain *C*. *udeis* LP-R2r showed the highest β-1,3-glucanase activity in both qualitative and quantitative assays and was significantly higher than all other bacterial strains. The ability to synthesize protease was observed in all strains except *P*. *myrsinacearum* HP-R1r (Tables 1 and 2). Strain *P*. *phytofirmans* LP-R1r showed the greatest protease activity in the plate assay (15–25 mm clear zone) and broth assay (82 units of protease enzyme produced per mL of broth). Cellulase activity was observed for all but one strain (*P*. *frederiksbergensis* HP-N1r) in plate-based and broth-based assays (Tables 1 and 2). Strains *C*. *udeis* LP-R2r and *P*. *phytofirmans* LP-R1r showed the greatest abilities to degrade cellulose as indicated by the clearance zone size around bacterial growth on plates and by the amount of cellulase enzyme produced per mL of broth. It is interesting to note that strains *C*. *sordidicola* HP-S1r, *P*. *phytofirmans* LP-R1r and *C*. *udeis* LP-R2r showed positive activity for all four cell wall degrading enzymes.

All bacterial strains were able to produce ammonia *in vitro* (Table 1). Particularly, strain *P*. *phytofirmans* LP-R1r showed the highest ammonia production of all strains, followed by strain *Pseudomonas mandelii* LP-S1r that showed medium ammonia production. Catalase enzyme activity was observed for all but one strain (*P*. *frederiksbergensis* HP-N1r), with strain *C*. *udeis* LP-R2r showing the highest catalase activity (Table 1).

## Discussion

In this study, our main motive was to investigate the PGP abilities of endophytic bacterial strains isolated from lodgepole pine trees growing in a nutrient-poor, disturbed ecosystem in the SBPSxc region in BC. When the six endophytic bacterial strains were analyzed for their potential to enhance tree growth via inoculation studies with their original host (lodgepole pine) and a foreign host (hybrid white spruce), it was observed that all strains were effective in significantly increasing the length (30–60%) and biomass (125–302%) of both tree hosts, 540 days after inoculation (Figs 1 and 2). Comparable growth promotion has been observed in previous greenhouse studies conducted for a similar time period, in which endophytic PGPB were inoculated into Pinaceae trees such as lodgepole pine, Douglas-fir, Scots pine, western red cedar and hybrid white spruce [11, 26, 49–52]. However, the growth promotion observed for our strains may be overestimated and should be interpreted with caution since the pine and spruce seedlings were grown under sterile conditions in a greenhouse set-up which is far from natural edaphic conditions.

Forming a close association with their host is a major strategy employed by PGPB to stimulate plant growth and health [53]. This was true for bacteria tested in this study since all strains had formed ten thousand to ten million colonies per gram tissue in the rhizosphere and internal root tissues of spruce and pine seedlings 540 days after inoculation (Fig 3). Internal stem colonization was also detected for all bacterial strains; however, needle colonization was observed for a subset of strains, which indicates niche preferences exerted by either the bacterium or the tree host [54]. The rhizospheric and endophytic population sizes observed in our study align with those observed for other PGPB in coniferous as well as deciduous tree species [26, 32, 55–58]. However, plant colonization by our bacterial strains may vary significantly in natural field conditions when faced with significant abiotic and biotic stresses. Entry to and survival in internal plant tissues is facilitated by the secretion of protease and cellulase enzymes since these enzymes can disintegrate and metabolize plant cell wall polymers, proteins and other organic compounds in the apoplast [59, 60]. Since all strains possessed the ability to secrete at least one of these enzymes (Tables 1 and 2), it can be suggested that internal tissue colonization may have resulted due to the functioning of these enzymes. It is interesting to note that four strains that showed the presence cellulase and protease enzymes were the only ones that were able to colonize the needle tissues of both pine and spruce (Fig 3), potentially indicating the combined role of these enzymes in helping the bacteria to enter, move, survive and multiply in plants, particularly in the needle tissues.

The rhizospheric and endophytic bacterial population for each strain in pine and spruce had a strong correlation (R^2^ > 0.87) with seedling length and biomass (Fig 4). This suggests that the number of colonies formed by each bacterial strain directly affected their efficacy to enhance the growth of pine and spruce seedlings. Similar observations have been reported in inoculation studies with interior spruce, lodgepole pine, poplar, corn, canola and tomato [32, 56, 61–63]. The best plant colonizers in our greenhouse growth trials–*C*. *sordidicola* HP-S1r, *C*. *udeis* LP-R2r and *P*. *phytofirmans* LP-R1r –were the best plant growth promoters as they promoted seedling length by up to 60% and seedling biomass by up to 302% (Fig 4). However, it is difficult to determine whether this plant-growth-promotion effect was due to the rhizospheric population or endophytic population, or due to a synergistic effect of both populations, therefore further research focusing on this subject is necessary.

Phytohormones like IAA and ethylene play a crucial role in the growth and development processes of a plant such as seed germination, root development and proliferation, stem and root elongation, reproduction, and fruit ripening [23]. Modulation of these hormones by PGPB living in close association with the host plant is a well-known phenomenon to enhance the growth and health of the host plant in exchange for energy for the microbes [64]. For instance, when the endogenous production of IAA by plants is insufficient to support their growth and development, reliance on exogenous IAA produced by associative PGPB can be a viable alternative [28, 65]. Such PGPB convert L-tryptophan, a metabolite commonly present in plant exudates, into IAA indicating the development of a mutually-beneficial relationship between the PGPB and the host plant. The ability to convert L-tryptophan to IAA was confirmed in all of our strains via *in vitro* broth assay (Table 2). Since all strains were also observed to significantly enhance the length and biomass of pine and spruce seedlings in the greenhouse trials (Figs 1 and 2), our results are consistent with the theory that IAA production leads to greater elongation, proliferation and development of the plant tissues [23, 64, 66]. In particular, the highest IAA producing strains–*C*. *sordidicola* HS-S1r, *P*. *phytofirmans* LP-R1r and *C*. *udeis* LP-R2r –were also the best performing strains in the greenhouse which further support this theory. However, further evidence is required to confirm the IAA-producing ability of these strains *in planta* by raising IAA-negative mutants and then comparing the release of IAA in plant roots after inoculation with IAA-positive versus IAA-negative derivatives. In addition, *in planta* expression of genes related to IAA production such as *iaaH* and *iaaM* genes could be performed using qRT-PCR to detect the relevant gene copy numbers. The significant plant growth promotion observed specifically under nutrient-stress conditions in the greenhouse can also be linked to the ability of all of our strains to modulate plant-ethylene levels by releasing ACC deaminase (Table 2). Plants produce excess amounts of ethylene when subjected to stress conditions which can inhibit their growth and development [67]. However, certain associative PGPB produce ACC deaminase to cleave ACC–the precursor of plant-ethylene–and convert it to α-ketobutyrate and ammonia, thereby reducing excess plant-ethylene levels [23, 44]. ACC deaminase producing bacteria utilize the ammonia produced from this reaction for their own metabolism. We used a binary approach–*in vitro* enzyme assay and *in vivo* gnotobiotic assay–to evaluate the ACC deaminase activity of our bacterial strains. The amount of ACC converted to α-ketobutyrate by our bacterial strains (33–111 nmol/mg/hr) in the *in vitro* enzyme assay was analogous to the typical range observed in previous studies with endophytic bacteria [68–71]. All bacterial strains also showed positive ACC deaminase activity when inoculated into ethylene-sensitive plants–canola and tomato–in the *in vivo* gnotobiotic assay (Fig 5). It should be noted that a uniform and strong correlation (R^2^ > 0.75) between the *in vitro* and *in vivo* analyses of ACC deaminase activity was observed for our strains (Fig 6). The primary root length of inoculated canola and tomato seedlings was 2-fold or higher than non-inoculated control seedlings, which is consistent with the findings of Anandham et al. [72] and Onofre-Lemus et al. [73]. It has been postulated that alleviation of excess plant-ethylene levels after seed germination by ACC deaminase producing bacteria is a priming effect to significantly enhance the root length of inoculated seedlings after germination. Although excess ethylene levels are required to break seed dormancy, high levels after germination could lead to stunted growth. However, ACC deaminase producing bacteria could negate this effect [23, 44]. Although our strains showed significant results for *in vivo* root elongation–suggesting the synthesis of ACC deaminase enzyme–however, to confirm this, *in planta acdS* gene expression of these bacterial strains needs to be evaluated. Additionally, specific proteins secreted by each bacterial strain to cleave ACC need to be identified so as to confirm the secretion of these proteins *in planta* following inoculation.

Lytic enzymes, including cellulase, protease, chitinase and β-1,3-glucanase, represent a major category of secondary metabolites released by PGPB to lyse cell walls of plants and microorganisms as well as cuticles and eggshells of pests like nematodes which may lead to suppression of phytopathogens and/or decomposition of litter and nutrient turnover [74–76]. All of our strains were tested positive for the presence of at least one of these four enzymes in both qualitative and quantitative assays, with comparable results observed for each strain in both assay types. The enzyme units of cellulase, protease, chitinase and β-1,3-glucanase produced by our strains fall within the usual range detected for rhizospheric and endophytic PGPB [37, 77–80]. However, the enzyme activity detected under *in vitro* conditions should be interpreted with caution because the ‘actual’ production of these enzymes may vary under *in vivo* conditions where a variety of external factors can affect the syntheses of these enzymes. Methods such as *in situ* gene expression and proteomics needs to be applied to confirm the activity of these enzymes in real plant conditions. In addition, the antagonistic potential of these strains against plant pathogens must be evaluated through co-inoculation of these strains with a pathogen in plant to confirm if the production of these enzymes ‘actually’ leads to the suppression of the pathogen. Each lytic enzyme has a specific function to degrade particular compounds in the cell walls and cuticles. For example, cellulase enzyme degrades the glycosidic linkages in cellulose chains that form intra- and intermolecular hydrogen bonds, protease enzyme breaks the peptide bonds present in the protein matrix, chitinase enzyme disintegrates the rigid chitin polymer, and β-1,3-glucanase enzyme degrades the β-1,3-linked backbone of glucan, a cell wall polysaccharide [78]. Notably, the activity of all four enzymes was detected only in *C*. *sordidicola* HS-S1r, *P*. *phytofirmans* LP-R1r and *C*. *udeis* LP-R2r, which indicates that these strains may be effective at controlling phytopathogens and/or decomposition by possibly exerting a synergistic mechanism. Another common biocontrol mechanism includes the production of toxic compounds like ammonia gas by PGPB [81]. All of our strains possessed the ability to produce ammonia gas as observed by the formation of yellow/brown colour when Nessler’s reagent–a common ammonia-detecting compound–was added to the broth. In comparison to other strains, the development of darker brown colour for *P*. *phytofirmans* LP-R1r indicated the highest ammonia production by this strain. All bacterial strains except *P*. *frederiksbergensis* HP-N1r were observed to secrete catalase (Table 1), which is an enzyme known to neutralize the overproduction of reactive oxygen species (ROS) under stress conditions. However, *in vitro* production of cellulase, protease, chitinase, β-1,3-glucanase and catalase enzymes cannot be related to their production in a plant system and further studies need to be conducted to observe the production of genes related to these enzymes via qRT-PCR. In addition, proteins relevant to these enzymes secreted by our strains need to identified, so that production of these enzymes could be confirmed in the plant-soil system after bacterial inoculation.

Siderophore production in combination with the ability to synthesize lytic enzymes and toxic gases is believed to be extremely lethal against phytopathogens, particularly fungi [82–84]. In addition to efficiently chelating the Fe^3+^ molecules present in soil and depriving the pathogens from taking up iron, siderophores produced by PGPB are also responsible for triggering ISR in plants [85–88]. All but one strain showed positive siderophore production in the range observed for PGPB isolated from natural and cultivated ecosystems [24, 26, 28, 82, 88, 89]. Along with assisting the plant in mediating biotic stress, PGPB-produced siderophores can also help the plant in acquiring ferric iron, a scarce micronutrient that mostly exists in plant-unavailable form in soils [90]. PGPB can also facilitate plant acquisition of macronutrients like phosphorus via two major mechanisms–solubilization of inorganic phosphorus and mineralization of organic phosphorus [91]. Since the scarcity of plant-available phosphorus in soils is often an issue in both agriculture and forest ecosystems, the presence of PGPB that can effectively convert the unavailable forms of phosphorus in the soil to available forms is vital for sustainable productivity [23]. *In vitro* plate and broth assays revealed that all but one bacterial strain possessed both inorganic phosphate solubilizing and organic phytate hydrolyzing abilities (Tables 1 and 2). For inorganic phosphate solubilization, the SI observed in the plate assay (1.2–2.6) and the amount of soluble phosphates released in the broth assay (66–112 μg per mL broth) by our bacterial strains align with previous studies in which PGPB isolated from crop and tree hosts with strong phosphate solubilization abilities have been reported [24, 26, 28, 37, 92–94]. The mechanisms of inorganic phosphate solubilization mainly include the production of organic acids, protons, siderophores and exopolysaccharides [91]. Although several organic acids could be released by PGPB, gluconic acid represents one of the most common and effective forms of organic acid responsible for releasing phosphorous [95]. Since the major source of phosphorus in forest ecosystems is organic litter, the ability to produce phytase to mineralize certain forms of organically-bound phosphorus is crucial for the growth of trees. As plants are not known to directly take up phytate from the soil or mineralize it, phytase-secreting microbes have an important role to play in hydrolyzing phytate and making it available for plant uptake [91, 96]. The five strains that hydrolyzed Na-phytate in both qualitative plate assay and quantitative broth assay (Tables 1 and 2) performed similarly well in comparison to previous studies [38, 39, 96, 97]. All in all, the five strains that possess the ability to make both inorganic and organic phosphorous available for plants could be referred to as comprehensive phosphate solubilizing bacterial strains. In particular, *C*. *udeis* LP-R2r which had significantly higher inorganic and organic phosphate solubilizing ability than all other strains should be further evaluated to quantify the amount of phosphorus made available by this strain *in planta*. Furthermore, additional data needs to be collected to confirm the increased phosphorus uptake by plants when inoculated with these strains using stable isotope method, gene expression analysis and proteomics.

Since both lodgepole pine and hybrid white spruce are conifer species that have been reported to associate widely with mycorrhizal fungi in natural temperate and boreal forests, it is inevitable to sideline their role in tree growth promotion. In a previous study, lodgepole pine and other Pinaceae tree species including interior Douglas-fir (*Pseudotsuga menziesii*) and ponderosa pine (*Pinus ponderosa*) inoculated with isolates of *Laccaria laccata*, *Rhizopogon vinicolor* and *Suillus luteus* showed a significant increase in height (up to 23%) and root collar diameter (up to 45%), 2 years after inoculation [98]. Intriguingly, authors confirmed that the observed growth responses of conifer seedlings were partially influenced by IAA and ethylene produced by these ectomycorrhizal fungal symbionts [98]. In another study, isolates of ectomycorrhizal fungus *Hebelomaarenosa arenosa* were reported to enhance the shoot height and root dry weight of red pine (*Pinus resinosa*) when grown without fertilizer applications in a greenhouse [99]. These fungal isolates also increased the survival rate of red pine seedlings after outplanting into field conditions. Similarly, above-ground growth of radiata pine (*Pinus radiata*) seedlings was significantly enhanced by inoculation with *Rhizopogon roseolus* and *Scleroderma citrinum* isolates, 2 years after outplanting onto low soil moisture sites in Spain [100]. It is interesting to note that ectomycorrhizal fungi and PGPB have been observed to work synergistically to promote the growth of lodgepole pine and hybrid white spruces seedlings in greenhouse studies indicating that their combined capability could be sufficient to support the growth of Pinaceae trees on disturbed sites [101, 102]. Therefore, our PGPB strains should be evaluated with mycorrhizal fungi in future field-based studies to determine their ‘real’ benefits and evaluate if they work coherently with mycorrhizal fungi to sustain the growth of Pinaceae trees in the SBPSxc region.

In conclusion, the six bacterial strains evaluated in this study can colonize the rhizosphere and internal tissues of multiple Pinaceae trees–lodgepole pine and hybrid white spruce–and enhance their growth by potentially exerting diverse PGP mechanisms. Of the 11 mechanisms tested in this study, all bacterial strains were tested positive for at least 5 different mechanisms involving key PGP traits. Notably, three bacterial strains–*C*. *sordidicola* HS-S1r, *P*. *phytofirmans* LP-R1r and *C*. *udeis* LP-R2r –possess the highest potential to promote plant growth using all mechanisms tested in this study. This impressive suite of *in vitro* PGP capabilities could be related to the significant enhancement of pine and spruce length and biomass observed for these strains in the greenhouse growth trial. Bacterial strains belonging to *Caballeronia* and *Paraburkholderia* genera possess multiple PGP abilities as reported in previous studies using lab-based enzyme assays, genomic analyses, and *in planta* assays [9, 33, 103–105]. Intriguingly, these genera were previously assigned to the plant-beneficial group of the *Burkholderia* genus [106, 107], which is rich in potent plant-probiotics [21, 108]. Although we have demonstrated the growth-promoting potential of our strains in the greenhouse experiment, the *in vitro* PGP experiments do not provide enough evidence for the mechanisms behind the observed growth promotion. The *in vitro* conditions in which the PGP abilities of our bacterial strains were observed represent highly sterile and simulated conditions, therefore functioning of these PGP mechanisms must be validated under realistic conditions *in planta*. Potential approaches may include raising negative mutants lacking the relevant PGP trait and comparing it with the wild-type strain via plant inoculation studies, conducting whole genome sequencing of these bacterial strains to identify their PGP-related genes and then evaluating the expression level of PGP-related genes *in planta* following inoculation, and co-inoculation of plant with a pathogen and our PGP strains to observe their ability to secrete various lytic enzymes and suppress pathogen in natural conditions. Considering the results of this study and a previous report where these bacterial strains showed considerable nitrogen-fixing ability [5], we can conclude that such strains with multifarious PGP abilities may be playing a significant role in sustaining the growth of Pinaceae trees under nutrient-limited, disturbed edaphic conditions of the SBPSxc region. Henceforth, such bacteria with the inherent ability to enhance the growth of multiple tree hosts should be further evaluated in the field to determine their actual benefits under natural conditions.
